# Creatine Kinase Elevations and Risk of Renal Failure and Dialysis in Patients With Rhabdomyolysis

**DOI:** 10.7759/cureus.86536

**Published:** 2025-06-22

**Authors:** Dietrich V Jehle, Heidi Schultz, Rochak Khatri, Paul J Forks, Krishna K Paul, Shelby A Reed, Olu Akinwande, Hailey P Hoffmann, Danielle O'Connell

**Affiliations:** 1 Department of Emergency Medicine, University of Texas Medical Branch at Galveston, Galveston, USA

**Keywords:** acute kidney injury, creatine kinase, dialysis, renal failure, rhabdomyolysis

## Abstract

Background

Rhabdomyolysis is characterized by muscle necrosis, which releases creatine kinase (CK) into the circulation. Acute kidney injury (AKI) is a common complication resulting from renal ischemia, tubular obstruction, and tubular injury due to heme pigments. While a correlation between AKI and CK levels has been reported previously, the precise relationship is unclear. This study examines the relationship between CK levels and outcomes of renal failure and dialysis within seven days of rhabdomyolysis diagnosis.

Method

This retrospective study utilized the TriNetX database to evaluate 93,479,176 patients from 56 healthcare organizations in the United States from 2003 to 2023. A total of 73,036 adults ≥18 years old were identified with a CK value ≥ 1,000 U/L within three days after a rhabdomyolysis diagnosis. Patients were further stratified based on CK values (1K-5K, 5K-10K, 10K-50K, 50K-100K, and >100K). Exclusion criteria included a rhabdomyolysis diagnosis more than 20 years prior, history of dialysis, and renal failure (Cr ≥4). Descriptive statistics and relative risks of dialysis and renal failure were calculated for each subgroup compared to those with CKs between 1K and 5K (control).

Results

There was a statistically significant increase in risk of dialysis and renal failure (Cr ≥4) for each progressive cohort compared to the control group, with CK 1K-5K (Dialysis 1.84%, Cr ≥4 6.38%), 5K-10K (2.88%, 7.99%), 10K-50K (5.02%, 13.73%), 50K-100K (8.79%, 21.01%), and >100K (8.81%, 24.97%), respectively.

Conclusions

This is the largest study to date evaluating the association between CK levels and rhabdomyolysis severity. With each progressive increase in CK, the risk of renal failure (CR ≥4) and the need for dialysis also increased.

## Introduction

Rhabdomyolysis is a pathological condition characterized by myocyte damage and the subsequent release of electrolytes and intracellular molecules, including creatine kinase (CK) and myoglobin, into circulation. This release can lead to significant downstream effects [1-6]. Rhabdomyolysis can result from various causes, most notably trauma, but also from toxins, infections, metabolic disorders, and ischemic events. Often, the condition is multifactorial [1]. Regardless of its cause, common symptoms include muscle pain, weakness, and dark urine. An elevated CK level, indicative of muscle degradation, is a key diagnostic marker of the disease [1].

A major concern with rhabdomyolysis is its impact on renal function. Alarmingly, up to 50% of patients with rhabdomyolysis develop acute kidney injury (AKI), contributing to 5-25% of all AKI cases [2,3]. AKI involves a sudden decline in kidney function, reducing the ability to filter waste products. Consequences include electrolyte imbalances, fluid retention, and volume overload [4]. AKI can lead to multi-organ dysfunction, making prompt recognition and treatment essential. While increased creatinine levels are a hallmark of AKI, serum CK levels are also critical in assessing kidney injury in rhabdomyolysis, often rising more than 10 times above the normal upper limit of approximately 200 U/L [5]. Elevated CK levels provide insights into the severity of rhabdomyolysis and its impact on the kidneys [7].

Due to the severe implications of rhabdomyolysis and AKI, it is essential to detect and treat these conditions appropriately. Although previous studies have shown a correlation between CK levels and kidney injury, the exact nature of this relationship and its variability among individuals is not fully understood. Variability in CK levels suggests that they may not be sufficient alone to predict AKI reliably [6]. Furthermore, past research has been limited by small sample sizes and narrow geographic scopes [1,3,6].

Study objectives

This study aims to evaluate the relationship between CK levels and the risk of dialysis and severe renal failure in a retrospective cohort of approximately 73,000 patients diagnosed with rhabdomyolysis from the TriNetX database.

## Materials and methods

Data source and study population

TriNetX is a global, federated healthcare research network that allows access to the electronic medical records (EMR) of large healthcare organizations (HCOs). This report was a retrospective, observational study on data from 56 HCOs with 93,479,176 patients in the US Collaborative Network within the database. Data was collected based on International Classifications of Diseases 10th revision (ICD-10-CM) codes for diagnosis [8], Logistical Observation Identifiers Names and Codes (LOINC) for labs [9], and Current Procedural Terminology (CPT) and Healthcare Common Procedure Coding System (HCPCS) [10] for procedures.

Cohort selection

Patients were included if they were adults ≥18 years old with a diagnosis of rhabdomyolysis within 20 years of May 24, 2003, to May 24, 2023. The diagnosis of rhabdomyolysis was determined by ICD-10-CM M62.82 - “Rhabdomyolysis”. Qualifying patients’ data were separated into cohorts based on their lab CK values tested within three days of or after the diagnosis of rhabdomyolysis. The values were: greater than 1000 U/L, 1000-5000 U/L, 5000-10000 U/L, 10000-50000 U/L, 50000-100000 U/L, and greater than 100000 U/L. These rounded cutoffs were chosen due to clinical relevance and for ease of interpretation [2,11,12]. The cohort defined as “greater than 1000 U/L” is meant to represent the average findings for all ranges in the study. Patients' lab CK values were queried with the code LOINC 2157-6. Exclusion criteria for the derived study population were patients who were diagnosed with rhabdomyolysis more than 20 years ago, younger than 18 years old at age of diagnosis, and prior history of receiving dialysis at least one day preceding the diagnosis of rhabdomyolysis, indicated by the procedure codes: CPT 1012740, ICD-9-CM 39.95, CPT 1012757, or HCPCS G0257.

Outcomes and statistical analysis

For each CK range cohort previously defined, the number of patients who encountered specific outcomes was identified and used for further calculations. Outcomes included dialysis (as defined previously) and creatinine greater than or equal to 4 mg/dL (Cr≥4) as a marker for renal failure within seven days after the rhabdomyolysis diagnosis. The value of Cr≥4 was chosen as this signifies seriously impaired renal function, often indicating the need for dialysis within the next year, as well as being commonly associated with anion gap metabolic acidosis [13,14]. Creatinine labs were identified by the code LOINC 2160-0. Patients who developed the outcomes prior to the time window were excluded from the results.

The risk for the outcomes was calculated within the database for each cohort. The risk was defined as the percentage of patients in the cohort who experienced the outcome within the time window compared to the total number of patients in the cohort. A chi-square test was performed between groups with a significance level defined as p<0.05. All outcome statistics were calculated through the Measure of Associations tool, which is embedded within the TriNetX database.

Disclaimer

Utilization of data from TriNetX did not require UTMB IRB review, as this was a secondary analysis of de-identified data. The UTMB IRB determined that this project is considered “not human subjects research”.

## Results

Patient characteristics

**Figure 1:**
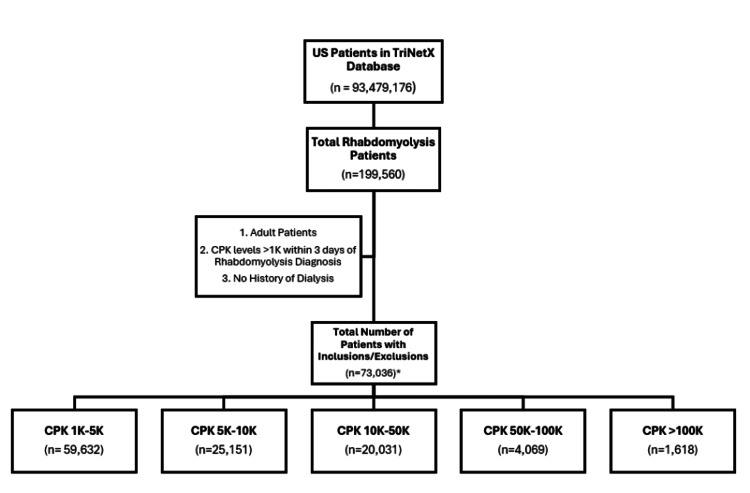
Cohort Selection Flow Chart CPK, Creatine phosphokinase *There may be patients with multiple measurements of CPK that belong to several subgroups.

The total number of rhabdomyolysis patients in this database was 199,560. After applying inclusion and exclusion criteria, our total population count was 73,036 (Figure 1). In this study, 70% of the patient population was male and 30% female. The mean age of patients was 57 years with a standard deviation of 19 years; the ages ranged from 18 to 90 overall. Seventy-three percent of patients reported their ethnicity as not Hispanic or Latino, 22% were of unknown ethnicity, and only 5% were Hispanic or Latino. The racial composition of the population was majority white at 59%, followed by 26% Black or African American, 13% unknown race, 1% American Indian or Alaskan Native, and 1% Asian. 

Common comorbidities to rhabdomyolysis experienced by the selected patients included disorders of fluid, electrolyte, and acid-base balance (63% of patients), acute kidney failure (57%), and essential (primary) hypertension (56%).

Descriptive results

**Figure 2:**
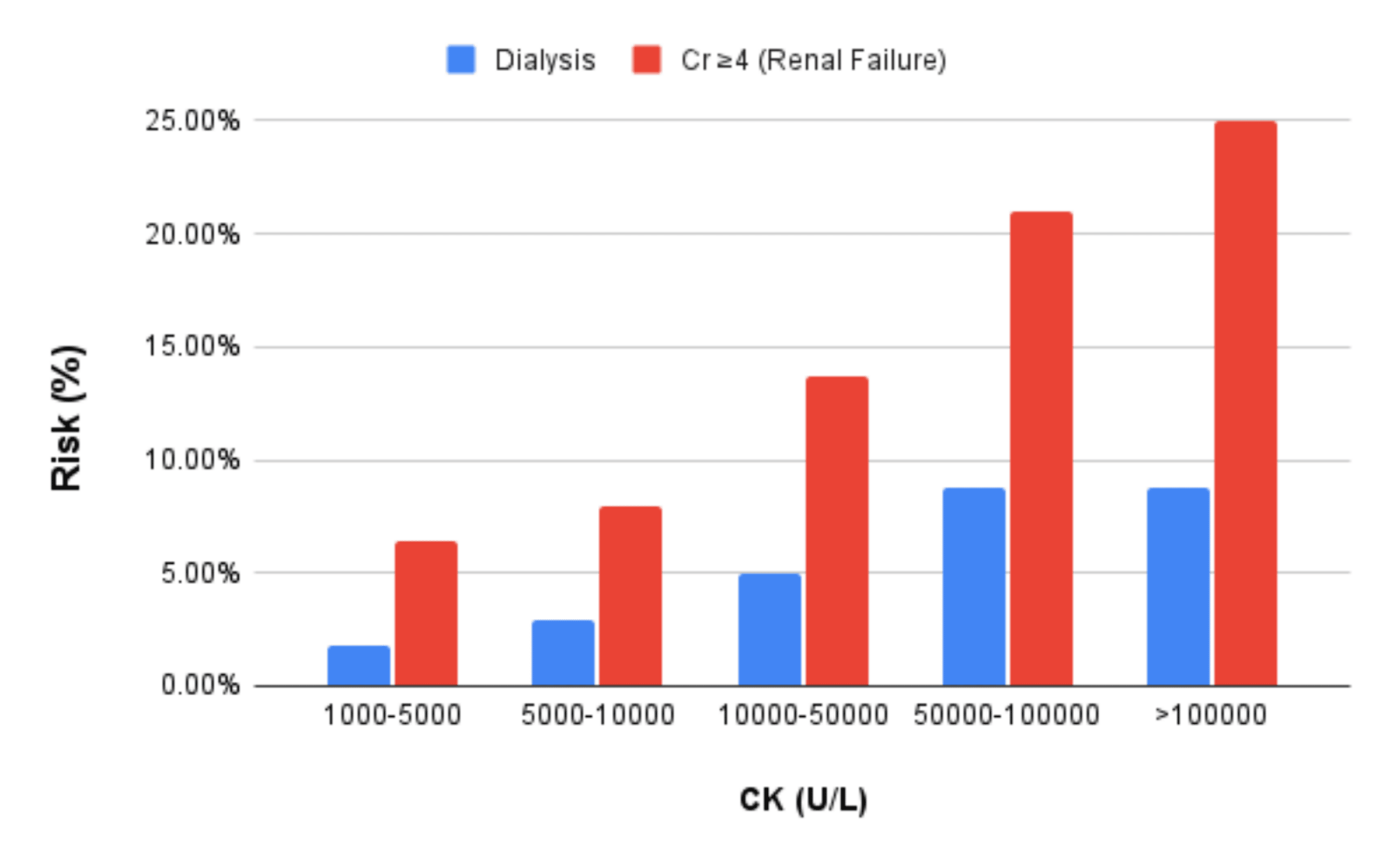
Outcomes for Rhabdomyolysis Patients CK, Creatine kinase

The overall trend of the findings was that risk in developing a particular outcome increases as the CK range increases. Additionally, the increase in risk for hemodialysis between the 50000 and 100000 U/L cohort and the >100000 U/L cohort was not as large as the increase in risk for the other two outcomes in the same cohorts (Figure 2).

Analytical results

When comparing each progressive CK range with a control group of CK 1K-5K, there was a statistically significant (p<0.001) increase in risk of dialysis and renal failure for each cohort: CK 1K-5K (Dialysis 1,094 (1.84%), Cr ≥4 3,572 (6.38%)), 5K-10K (717 (2.88%), 1,839 (7.99%)), 10K-50K (996 (5.02%), 2,539 (13.73%)), 50K-100K (350 (8.79%), 772 (21.01%)), and >100K (140 (8.81%), 379 (24.97%)), respectively (Tables 1, 2).

**Table 1:** Risk of Hemodialysis at Varying Creatine Kinase Levels in Rhabdomyolysis CK, Creatine kinase; U/L, Units of enzyme activity per liter; mg/dL, Milligrams per deciliter; K, x1,000; RR, Risk Ratio; 95% CI, 95% Confidence Interval Statistical analysis used was the chi-square test; p-value is considered statistically significant if <0.05

CK Value (U/L)	Patients Evaluated	Risk of Hemodialysis (%)	RR (95% CI)	Chi-Square	p-Value
5K-10K U/L	24,928	717 (2.88%)	1.56 (1.42-1.71)	89.49	p<0.001
1K-5K U/L	59,409	1,094 (1.84%)			
10K-50K U/L	19,826	996 (5.02%)	2.73 (2.51-2.97)	586.16	p<0.001
1K-5K U/L	59,409	1,094 (1.84%)			
50K-100K U/L	3,984	350 (8.79%)	4.77 (4.25-5.36)	808.70	p<0.001
1K-5K U/L	59,409	1,094 (1.84%)			
>100K U/L	1,590	140 (8.81%)	4.78 (4.04-5.66)	378.85	p<0.001
1K-5K U/L	59,409	1,094 (1.84%)			

**Table 2:** Risk of Renal Failure with Creatinine ≥4 mg/dL at Varying Creatine Kinase Levels in Rhabdomyolysis CK, Creatine kinase; U/L, Units of enzyme activity per liter; mg/dL, Milligrams per deciliter; K, x1,000; RR, Risk Ratio; CI, Confidence Interval Statistical analysis used was the chi-square test; p-value is considered statistically significant if <0.05

CK Value (U/L)	Patients Evaluated	Risk of Creatinine ≥4 mg/dL (%)	RR (95% CI)	Chi-Square	P-Value
5K-10K U/L	23,008	1,839 (7.99%)	1.25 (1.19-1.32)	66.97	p<0.001
1K-5K U/L	56,034	3,572 (6.38%)			
10K-50K U/L	18,497	2,539 (13.73%)	2.15 (2.05-2.26)	998.59	p<0.001
1K-5K U/L	56,034	3,572 (6.38%)			
50K-100K U/L	3,675	772 (21.01%)	3.30 (3.07-3.54)	1,094.55	p<0.001
1K-5K U/L	56,034	3,572 (6.38%)			
>100K U/L	1,518	379 (24.97%)	3.92 (3.57-4.30)	799.045	p<0.001
1K-5K U/L	56,034	3,572 (6.38%)			

## Discussion

In this retrospective analysis, it was found that higher CK levels correlate with increased clinical severity of rhabdomyolysis, specifically for hemodialysis and outcomes of severe renal failure, represented by Cr≥4 mg/dL. While other studies have examined CK as a prognostic marker, this study is the first to do so with such a large sample size (n>73,000). Although there is widespread agreement that CK is a valuable and sensitive marker for diagnosing rhabdomyolysis, its usefulness as a marker of severity is less clear. Some studies suggest that CK is weakly predictive of prognosis [1,15,16], while others find it has little to no predictive value [6,17,18]. However, these studies often involve smaller sample sizes and inconsistencies in measuring the clinical severity of rhabdomyolysis. Given the diverse etiologies of rhabdomyolysis, it is crucial to study a representative population to apply findings clinically. Our study demonstrated that CK is a useful marker for identifying the severity of this condition, as shown by its association with elevated creatinine levels and the need for hemodialysis in a large and robust sample population.

Previous studies support our finding that increased CK is associated with a higher risk of elevated creatinine levels [1,19]. This is expected, as CK levels reflect the degree of muscle necrosis, and higher values likely indicate a greater risk of renal injury. Although the predictive value of CK for negative outcomes has been ambiguous, it is well established that elevated creatinine levels are associated with renal injury and an increased risk of renal failure [18]. The ambiguity might stem from inconsistent definitions of renal injury, as few studies examine the relationship between CK and markers of renal injury beyond the need for hemodialysis.

Another notable finding of this study is that patients with elevated CK levels were more likely to require hemodialysis. This outcome is consistent with previous research [6]. While some studies have identified admission creatinine as the most significant predictor for hemodialysis need, combining CK measurement with creatinine levels may improve the accuracy of patient prognosis [20].

Limitations

One limitation of this study is its retrospective design, which shows association but limits the ability to imply causation. While other studies have also investigated CK values as a predictor of clinical severity of rhabdomyolysis [7,21], this study contains a substantially larger sample size allowing for more accurate generalization. Although this study was carried out in over 56 separate HCOs, we are unable to evaluate the clustering of sites or specific hospital distribution due to privacy policies of TriNetX. This can introduce bias as different regional epidemiology risk factors associated with poorer prognosis cannot be accounted for in our data analysis. A weakness inherent to studies based on electronic health records are limitations of classification due to coding errors. This study did not adjust for demographics, comorbidities, timing of intervention, heterogeneity of rhabdomyolysis etiologies, or factors associated with increased need for dialysis such as severe sepsis or nephrotoxic drug use, which may confound these results. Additionally, some patients belong to multiple cohorts due to their CK levels being collected across different ranges at various times during the study. It is worth noting that the number of patients in each cohort decreases as CK levels increase, which may affect data interpretation. This reduction in the cohort size at higher CK ranges limits statistical power and may introduce selection bias, as patients with extreme values likely represent a more clinically severe subgroup.

## Conclusions

This study demonstrates that CK may serve as an effective biomarker for predicting patients at risk of developing renal failure, specifically those with creatinine levels ≥4 mg/dL or requiring hemodialysis. These findings enable more accurate triage and identification of patients at elevated risk for renal injury and rhabdomyolysis, reinforcing the clinical utility of CK as a biomarker of disease severity and informing potential improvements in treatment strategies. Future research should explore the role of CK as an independent predictor of rhabdomyolysis, stratified by etiology, and identify risk factors contributing to increased rates of renal failure in affected patients.
